# Government’s impression management strategies, trust in government and social cohesion: An evidence from Songjiang University Town, China

**DOI:** 10.3389/fpsyg.2022.951579

**Published:** 2022-09-07

**Authors:** Juan Fan, Wenhui Liang, Hanyi Zheng

**Affiliations:** ^1^School of Journalism and Communication, Shanghai International Studies University, Shanghai, China; ^2^School of Literature and Journalism, Sichuan University, Chengdu, Sichuan, China

**Keywords:** promotive impression management, protective impression management, trust in government, social cohesion, political loyalty

## Abstract

Trust in government and social cohesion are crucial guarantees for long-term social stability. With the development of the Internet, cross-border flows of information have become increasingly easier, enabling more factors to influence people’s political perceptions and loyalty. This study explores the mechanism of governments’ impression management behaviors on trust in government and social cohesion using the questionnaire survey with college students in Shanghai as the research subjects. Impression management strategies are classified into promotive ones and protective ones herein based on the social psychology theory. The results indicate that both promotive and protective impression management strategies take by governments positively affect citizens’ loyalty, and loyalty positively affects citizens’ social cohesion and the level of trust in government; moreover, the level of trust in government positively affects social cohesion. These findings provide implications for boosting the sound development of China’s political society during the transition period.

## Introduction

As the global economy, culture and information networks become increasingly connected in this new era, exchanges and integration across countries are ever deepening, accompanied by drastic collisions and games of different ideologies. Against this backdrop, all countries around the world attach great importance to the build-up of social cohesion among the public (Huang, 2015). For instance, Belgium has issued stamps themed on social cohesion; the United States seeks to enhance its overall social cohesion by fostering a mainstream culture centered on individual freedom and a political culture centered on the recognition of civil rights; and Japan strives to unite the public through vigorous economic development and social mobilization.

It has been observed that the higher the recognition of government behaviors by the public, the stronger the overall social cohesion (Huang, 2015). According to the fragile state index in 2014, China was 79.0, ranking 68 in the world, which was a high warning, of which the government performance score was 7.9. It can be seen from this that the performance of the Chinese government needs to be strengthened (Carlsen and Bruggemann, 2017). However, through promotive and protective impression management strategies, governments can more truthfully and accurately convey information about the work of government departments to the masses, thus establishing themselves as authoritative governments with credibility. The increasingly approachable government image and transparent government behaviors highlight that the credibility and authority of local governments is imperative to foster “social cohesion” (Huang, 2015).

Then, how does governments’ impression management affect social cohesion? What role does trust in government play in this relationship? To address these questions, this study has examined the mediating role of citizens’ loyalty in the relationship between impression management and trust in government and social cohesion using the questionnaire method, explored the relationship between trust in government and social cohesion, and tested the proposed hypotheses using the SmartPLS statistical analysis software.

### Model construction and research hypothesis

Since the 1980s, a host of international researchers on Chinese issues have employed the “authoritarianism” paradigm to interpret China’s politics. However, as China’s politics evolves, such “authoritarianism” paradigm has become no longer sufficient to account for the changes taking place in China’s politics and society, such as Beijing’s adoption of opinions from a plurality of subjects and a greater focus on performance in its policy making (Lieberthal and Oksenberg, 1988). Based on the review of literature on the impression management theory (Goffman, 1959), trust in government (Miller, 1974; Hetherington, 1998), and social cohesion, this study constructs a theoretical model of impression management - trust in government - social cohesion.

### Governments’ impression management and political loyalty

Impression management refers to the maintenance or improvement of an organization’s image in the minds of its stakeholders through information communication and interaction (Leary and Kowalski, 1990).

Different from propaganda, government impression management can be divided into promotive impression management and protective impression management. The former refers to the behavior of trying to make others see their efforts positively, including strategies such as self-promotion, strengthening interaction and model setting. The latter refers to defensive measures that try to weaken their own shortcomings or avoid making others view themselves negatively, including statements and explanations (Yang, 2014). The strategy of promotive impression management can show a positive image to the audience, while the strategy of protective impression management can convey the feeling of depression and vulnerability to the audience, and the propaganda is often positive (Tedeschi, 1990).

Several studies in recent years have observed that, during the economic and social transition period in China, local government leaders exhibit extensive behaviors aimed at managing their images (or impressions) in the minds of their stakeholders (Yan and Wu, 2013). There are two main mechanisms by which governments’ impression management behaviors may boost the public loyalty to the country:

#### First, social expectations

Governments behave in line with the expectations of society as well as social norms. Local governments actively promote the modernization of their governance systems and governance capabilities, and the public’s sense of achievement rises substantially amidst common construction and shared development. Meanwhile, governments actively deepen reforms to continuously promote social justice, shaping effective social governance. Immersed in such conditions, the public can be greatly triggered to develop their political loyalty (Yu, 2019).

#### Second, relationships

Government officials represent the image of governments to a large extent. In the process of communication and interaction with the public, government officials often shape the first impressions by delivering intuitive feelings and tangible experiences to the public. If such impressions are damaged, the relationships between governments and the public will be distorted or interrupted (Zhao, 2008). Therefore, proper impression management behaviors can improve the relationships between governments and the public, which in turn boosts the loyalty of the public. Thus, the following hypotheses are proposed:

*H1: Governments’ promotive impression management positively affects the political loyalty of the public, i.e., the stronger the promotive impression management behavior, the higher the loyalty, and vice versa.*

*H2: Governments’ protective impression management positively affects the political loyalty of the public, i.e., the stronger the protective impression management behavior, the higher the loyalty, and vice versa.*

### Political loyalty, trust in local government, and social cohesion

In civilized societies, no quality is more important than loyalty, and no virtue is more valued by the states, classes, or political parties than loyalty (Zuo and Tu, 2018). Loyalty, as a human emotion and a strong expression of emotions, represents a kind of emotional attachment to a social group rather than an expression of rationality in the first place. Loyalty is closely related to behaviors of individuals; it is more of an emotion than a cognition. Another scholar, however, has suggested that loyalty is by no means merely an emotion, but is subject to self-control (Connor, 2007). From the above two opposing views, loyalty is first a positive moral emotion. However, it is not just an ordinary emotion. Only when a loyalist can choose the object of loyalty can the value of loyalty be judged.

Trust has been defined as “a psychological state composing the intention to accept vulnerability based on expectations of the intentions or behavior of another” (Rousseau et al., 1998). Trust is an important construct catalyst in many transactional relationships (Kassim and Asiah, 2010). There are also many studies, especially brand marketing studies, which have confirmed the correlation between trust and loyalty (Nguyen et al., 2014). Trust is acknowledged as an important indicator in developing customer loyalty. Similarly, the higher the loyalty, the higher the customer’s trust in the brand (Castaneda, 2011).

Social cohesion affects the performance of nations, and it refers to the “social contract” or interpersonal relationship between major groups-business, labor, government, and citizens (McCracken, 2003). In the 1990s the concept of social capital defined here as the norms and networks that enable people to act collectively—enjoyed a remarkable rise to prominence across all the social science disciplines (Woolcock and Narayan, 2000). Stanley (2003) believes that social cohesion refers to the willingness of social members to cooperate with each other for survival and prosperity. A society is “cohesive” if it is committed to the well-being of all its members, opposes exclusion and marginalization, creates a sense of belonging, promotes trust, and provides opportunities for upward mobility for its members (OECD, 2012). Different researchers and social scientists have slightly different views on social cohesion, but most scholars believe that it can be regarded as a kind of solidarity phenomenon in society (Pervaiz and Chaudhary, 2015). Unfair phenomenon will lead to social exclusion, and then reduce social cohesion (Van Staveren and Pervaiz, 2017).

Political loyalty, defined as a political virtue, and based on a certain political relationship or belief, implies individuals’ identification with and dedication to political causes, beliefs, ideals, and principles (Zuo, 2010). Political loyalty influences trust in government and social cohesion from the following two aspects: First, individuals with higher political loyalty are more likely to internalize the core values propagated by their governments. The core socialist values of China’s citizens take patriotism as the cornerstone. The higher the degree of political loyalty, the higher the degree of identification with the core values propagated by governments, and accordingly, the higher the degree of internalization, and the stronger individuals’ trust in governments and social cohesion. Second, national strength and development is a key source of public honor. Individuals with higher political loyalty have stronger sense of national honor and thus stronger individual trust in government and social cohesion. As such, the following hypotheses are proposed herein:

*H3: Political loyalty positively affects social cohesion, i.e., the higher the loyalty of citizens, the stronger the social cohesion, and vice versa.*

*H4: Political loyalty positively affects trust in government, i.e., the higher the loyalty of citizens, the stronger their trust in local government, and vice versa.*

### Trust in local government and social cohesion

With the current intensified downward pressure on China’s economy and numerous difficulties facing its social development, trust in government has become a dynamic indicator for indirectly interpreting changes in social cohesion (Huang, 2015). This view is supported by the Research Report on Public Trust in Government (2014) released by the National Institute of Social Development, Chinese Academy of Social Sciences at the end of 2014. The findings show that public trust in government exhibits a significant positive correlation with social cohesion. This correlation is particularly prominent at the local government level. It is because local governments, as the window of government “image,” have more direct contact with citizens in the process of performing social management functions. Therefore, the higher citizens’ trust in local government, the stronger the social cohesion. Hence, the following hypothesis is proposed herein:

*H5: Trust in local government positively affects public social cohesion, i.e., the higher the trust in local government, the stronger the social cohesion, and vice versa.*

**FIGURE 1:**
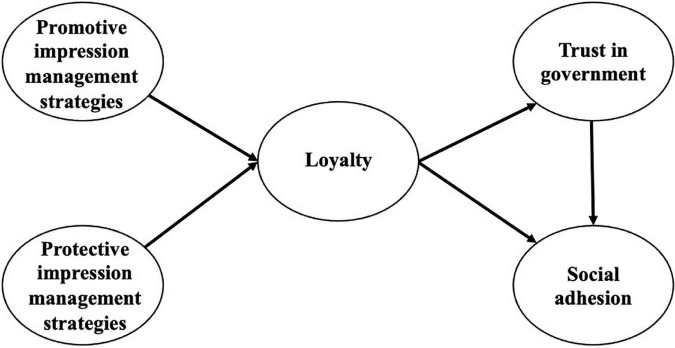
Research theoretical model.

Based on the above hypothesis derivation, the following theoretical model is proposed in this study, as shown in Figure 1.

## Materials and methods

The study adopted questionnaires as the primary research method, with college students as the surveyed subjects. The formal questionnaire survey was conducted in November 2018, and the questionnaires were distributed online to university students in the Songjiang University Town in Shanghai. A total of 390 questionnaires were collected, of which 336 were valid, representing an effective rate of 86%.

Specifically, the questionnaire consisted of two sections. The first section investigated demographic data, including gender, age, and education degree. The second section covered the research themes, including impression management strategies, loyalty, trust in local government, and social cohesion. The Likert 7 scale was employed, with scores of 1, 2, 3, 4, 5, 6, and 7 representing “strongly disagree,” “disagree,” “relatively disagree,” “neutral,” “relatively agree,” “agree,” and “strongly agree,” respectively. The main conceptual measures of the study are shown in Table 1.

**TABLE 1:** Research variables and corresponding indicators.

Variables	Indicators	Definitions of Indicators	References
Dependent variables	Trust in local government	Citizens’ belief or confidence that their local governments will operate to deliver results consistent with their expectations	
	Social cohesion	The degree of dependence, cooperation, and solidarity among the members of a society	Jenson, 1998
Independent variables	Promotive	A series of actions taken by an organization to promote its image	
	Protective	A series of actions taken by an organization to protect the actor from negative events	
	Political loyalty	Individuals’ identification with and dedication to political causes, beliefs, ideals, principles, etc.	

## Results

### Common method bias

To avoid the problem of common method bias, this study followed the recommendations of Podsakoff et al. (2003) by ensuring the anonymity of questionnaire completers and by distributing the independent and dependent variables separately, thus to procedurally minimize the common method bias.

The data were examined in this study. Harman’s single-factor ANOVA was employed (Podsakoff et al., 2003). The results from the unrotated factor analysis showed that the degree of explanation of the single factor was less than 40% (at 36.5%), indicating the absence of covariance between the independent and dependent variables (Doty and Glick, 1998; Fuller et al., 2016).

### Reliability test

Before analyzing the data, the study first examined the reliability of the survey data. Individual item reliability refers to the degree of reliability of a questionnaire, mainly in terms of consistency, coherence, reproducibility, and stability of the test results. By importing the research data into SPSS 26.0, the reliability of the scale of this study was tested, with the results shown in Table 2. As shown in the table, all indicators are above 0.7, indicating high consistency of the questionnaire scale.

**TABLE 2:** Reliability table for each construct in this study.

Sl. No.	Variables	Reliability values	Composite reliability
1	Promotive impression management strategies	0.77	0.84
2	Protective impression management strategies	0.79	0.89
3	Loyalty	0.83	0.87
4	Trust in government	0.89	0.87
5	Social cohesion	0.74	0.93

A scale is developed to measure a certain construct, and its ability to accurately measure that construct is referred to as its validity. According to the current United States Standards for Educational and Psychological Testing, validity can be divided into content validity, internal structural validity, and relational validity.

This study adopted the Expert Judgment Method (a.k.a. Delphi Method) to test the content validity. A questionnaire examination team was formed for the study, consisting of a professor of management, an associate professor of management, and two post-doctoral students of management. All these team members were engaged in the development and construction of the scales. Each measure was analyzed individually by the team members to determine whether the measurement entries were consistent with their perceptions of the construct. Controversial areas were then discussed, and agreement was eventually reached, ensuring the content validity of all scales in this study.

Factor analysis, the main tool for determining the internal structural validity, can be divided into exploratory factor analysis and confirmatory factor analysis. Prior to the exploratory factor analysis, this study first conducted a KMO test for each construct. Only constructs with KMO values greater than 0.6 were chosen for further factor analysis (Pallant, 2010). As shown in Table 3, the KMO values for the five constructs in this study are all greater than 0.6, justifying the performance of further factor analysis.

**TABLE 3:** KMO and Bartlett’s tests.

Constructs	KMO	Approx.χ^2^	Degree of freedom	Significance
Promotive impression management strategies	0.62	399.58	3	0.000
Protective impression management strategies	0.70	305.31	3	0.000
Loyalty	0.76	579.83	6	0.000
Trust in local government	0.82	838.432	6	0.000
Social cohesion	0.61	549.47	6	0.000

Table 4 shows the results of factor analysis of all the constructs herein, with the factor loading all exceeding 0.5, i.e., 0.55 as the minimum and 0.90 as the maximum, thus indicating good structural validity.

**TABLE 4:** Factor loading of all the constructs in this study.

Latent variables	Items for measurement	Factor loading
Social cohesion	I love my country very much.	0.85
	I am proud to be a Chinese citizen.	0.86
	I think that people in society are glad to help each other.	0.72
	I think that every member of society is equal.	0.55
Trust in local government	I think that the government is willing to listen to people’s opinions.	0.83
	I think that current policies are primarily for the benefit of the people.	0.87
	I think that the interests of the people can be effectively protected today.	0.88
	I think that the government handles things in a fair and appropriate way.	0.88
Loyalty	I won’t harm the interests of the country for my own interests.	0.87
	I won’t harm others’ interests for my own interests.	0.75
	I behave in accordance with social norms.	0.82
	I strive to behave in a way that maintains the image of the country.	0.83
Promotive impression management strategies	Government departments communicate care to the public.	0.90
	The publicity released by government departments to the public is vivid and approachable.	0.90
	Government departments send holiday wishes to the public during holiday seasons.	0.68
Protective impression management strategies	Government departments inform the public of risk factors in time.	0.83
	Government departments can clarify false information available in the society.	0.90
	Government staff will be publicly criticized and punished when they behave in a manner inconsistent with the government’s image.	0.78

The two most used indicators of relational validity are convergent validity and discriminant validity, which were first introduced by Campbell and Fiske (1959). Discriminant validity refers to a relatively low correlation coefficient of different traits measured by different methods. As seen from Table 5, the AVE value of each construct is greater than the covariance values between the constructs, and thus confirmed to have discriminant validity.

**TABLE 5:** Fornell-Lacker criterion.

Sl. No.	Variables	1	2	3	4	5
1	Social cohesion	0.75				
2	Loyalty	0.66	0.81			
3	Promotive impression management strategies	0.46	0.49	0.83		
4	Protective impression management strategies	0.23	0.41	0.54	0.84	
5	Trust in government	0.39	0.28	0.21	0.13	0.87

### Descriptive statistics

Table 6 shows that 52% of the samples studied were female, totaling 187 persons, and 48% were male, totaling 173. Among them, the largest age group was the group of 16 to 27 years old, of whom the group of 19 years old took the majority, totaling 84. Most of the respondents had an education level of bachelor’s degree, totaling 244 persons. As for the party membership distribution of the samples, 12.8% of them were party members while 86.9% of them were non-party members. As for the household income, the majority was concentrated in the range of 100,000–200,000 RMB, totaling 142 persons, followed by a total of 132 persons in the range of less than 100,000 RMB.

**TABLE 6:** Statistics of demographic variables.

Demographic variables	Options	Frequencies	Percentages
Gender	Male	173	48%
	Female	187	52%
Age	16	2	
	17	7	
	18	67	
	19	84	
	20	50	
	21	40	
	22	42	
	23	36	
	24	18	
	25	10	
	26	2	
	27	1	
Education level	Below bachelor’s degree	11	
	Bachelor’s degree	244	
	Master’s degree or above	105	
Political status	Party member		12.8%
	Non-party member		86.9%
Household income	Below 100,000	131	
	100,000–200,000	142	
	200,000–300,000	54	
	300,000–400,000	24	
	400,000–500,000	3	
	Above 500,000	6	

### Correlation analysis

Table 7 shows that governments’ promotive impression management strategies and protective impression management strategies were significantly and positively correlated with individuals’ political loyalty, trust in local government, and social cohesion; political loyalty was significantly and positively correlated with trust in local government and social cohesion; and trust in local government was positively correlated with social cohesion. These correlation analyses initially verified the hypotheses of this study, and a more precise verification of the influence mechanism between these variables was made later by regression analysis.

**TABLE 7:** Means, standard deviations and Pearson correlation coefficients of variables.

Sl. No.	Variables	Mean	S.D.	1	2	3	4	5
1	Promotive impression management strategies	6.11	0.80	1.00				
2	Protective impression management strategies	6.61	1.19	0.25**	1.00			
3	Political loyalty	6.17	0.71	0.45**	0.18**	1.00		
4	Trust in local government	5.03	0.94	0.23**	0.11*	0.28**	1.00	
5	Social cohesion	5.94	0.78	0.43**	0.12*	0.63**	0.40**	1.00

*n* = 336; **p < 0.01, *p < 0.05.

### Structural equation modeling

Structural equation modeling, as a crucial analysis method in social sciences, especially an important tool for multivariate data analysis, is a statistical method that analyzes the relationship between variables based on their covariance matrices. Due to the presence of multiple dependent variables in this study and the need to test for mediating effects, the structural equation model was chosen for estimation.

The research uses SmartPLS and partial least square method for statistical analysis. SmartPLS software is widely used in management, marketing, organizational behavior, information system and other fields. Partial least squares (PLS) is a new multivariate statistical data analysis method, which was first proposed by S. Wold and C. Albano in 1983. Partial least square method can realize regression modeling (multiple linear regression), data structure simplification (principal component analysis) and correlation analysis between two groups of variables (canonical correlation analysis) at the same time under one algorithm.

In this study, five variables, promotive impression management strategies, protective impression management strategies, loyalty, trust in government and social cohesion, were imported SmartPLS. Each latent variable corresponds to two or more observed variables. For example, promotive impression management strategies correspond to three observed variables: conveying care, self-propaganda and strengthening interaction; Protective impression management corresponds to three observed variables: informing risk, clarifying false information and public criticism strategy. Loyalty, social cohesion and government trust also correspond to four observed variables respectively.

First, we tested the fitting degree of the model. Chi-square is 368.62, and the chi square freedom ratio (CMIN/DF) is 2.95, which is between 1 and 3. It meets the fitting requirements of chi square freedom and passes the chi square test. The fitting index (GFI) is 0.92, greater than 0.9, and the TLI is 0.9, greater than 0.8, indicating that the initial model and sample data have acceptable fitness. In addition, the root mean square of residual error (RMSEA) is also the data that must be reported in the paper. It is not affected by the number of samples and the complexity of the model. It can estimate the statistical test power. The smaller the RMSEA index, the better the fitting degree of the model. According to the suggestions of Hu and Bentler (1999), if the RMSEA index is lower than 0.06, the model can be regarded as a poor model. If the RMSEA index is greater than 0.10, it means that the model is not ideal, 0.08 is the threshold for acceptable model fitting, and 0.05 means that the model fits well. Therefore, the RMSEA value of this study is 0.07, which meets the model adaptation standard.

**FIGURE 2:**
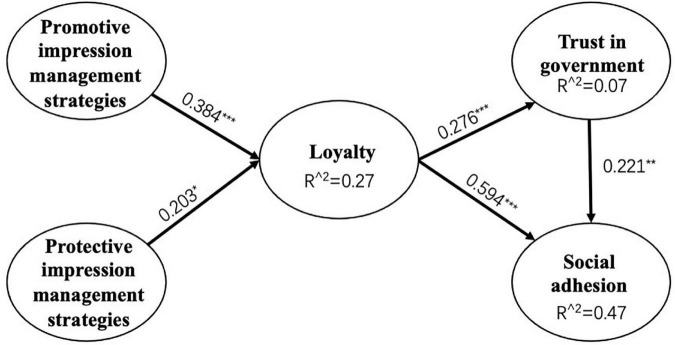
Diagram of model path coefficients.

Through model modification, we obtained the final diagram of model path coefficients. As shown in Figure 2, promotive impression management strategies positively affected public loyalty (β = 0.384, *p* < 0.001), thus H1 was validated; protective impression management strategies positively affected public loyalty (β = 0.384, *p* < 0.05), thus H2 was validated; loyalty positively affected social cohesion (β = 0.594, *p* < 0.001), thus H3 was validated; loyalty positively affected trust in local government (β = 0.276, *p* < 0.001), thus H4 was validated; trust in local government positively affected social cohesion (β = 0.221, *p* < 0.001), thus H5 was validated. A summary of the hypotheses validation in the study is shown in Table 8.

**TABLE 8:** Research hypotheses validation.

Hypotheses	Details	Validation status
**H1**	Governments’ promotive impression management positively affects the political loyalty of the public, i.e., the stronger the promotive impression management behavior, the higher the loyalty, and vice versa.	Validated
**H2**	Governments’ protective impression management positively affects the political loyalty of the public, i.e., the stronger the protective impression management behavior, the higher the loyalty, and vice versa.	Validated
**H3**	Political loyalty positively affects social cohesion, i.e., the higher the loyalty of citizens, the stronger the social cohesion, and vice versa.	Validated
**H4**	H4: Political loyalty positively affects trust in government, i.e., the higher the loyalty of citizens, the stronger their trust in local government, and vice versa.	Validated
**H5**	Trust in local government positively affects public social cohesion, i.e., the higher the trust in local government, the stronger the social cohesion, and vice versa.	Validated

## Discussion

The results of the data analysis indicated that both governments’ promotive impression management strategies and protective impression management strategies positively affected citizens’ loyalty, that loyalty positively affected social cohesion and the level of trust in local government, and that the level of trust in local government positively affected social cohesion. Thus, all the hypotheses of this study were validated.

The findings of the study provide implications for boosting the sound development of China’s political society during the transition period. Governments’ impression management behaviors not only exert direct positive effects on citizens’ trust in local government and social cohesion, but also generate indirect effects via the political and psychological variable of political loyalty. Hence, it is essential to take relevant measures to achieve information symmetry between governments and citizens, and regulate government behaviors.

First, it is necessary to change the traditional view of government performance, so that economic performance is no longer the sole criterion of success. Instead, citizens’ satisfaction toward their governments should also be taken into account, and the “people-oriented” service purpose should be specifically quantified as an indicator of government performance assessment (Han and He, 2006), thus to shape a “trustworthy” image of governments.

Second, the standard and capacity of local governments in the supply of public products and services should be upgraded. A pluralistic cooperation mechanism for public products and services should be set up to forge a model of joint social governance, striving to achieve information symmetry and meet diversified public demands.

Third, disinformation related to governments should be debunked promptly; acts that damage governments’ images should be punished in time to safeguard the positive images of governments. It is worth noting that during the implementation of impression management behaviors, governments should also be aware of the possible negative effects, which may weaken the public trust in government (Chen, 2018). Examples include the over-amplification of government performance and the evasion of crucial points while dwelling on the trivial when explaining negative information. Therefore, governments should never wear the wrong “mask” in their actions and maximize the positive effect of impression management.

In addition, it has also been observed that trust in local government positively affects citizens’ level of social cohesion. Government behaviors directly reflect the fulfillment of governmental functions and public service provisions, which thus provide the intuitive basis for citizens to evaluate their governments. The more government behaviors meet people’s expectations, the more it is conducive to enhancing citizens’ political trust and social cohesion.

Previous studies on impression management mostly focused on enterprise management. This study expands the application of impression management in political psychology, and tests the relationship among impression management, government trust and social cohesion by empirical methods, and builds a theoretical model of the three, which has theoretical significance. In addition, the study also puts forward suggestions on government impression management, which has practical significance for promoting the healthy development of China’s political society in the transition period.

Of course, the research also has obvious defects. To facilitate the collection of samples and ensure the quality of questionnaire filling, we selected the students at Shanghai Songjiang University town where the researcher is located as the research object, which has certain limitations. The follow-up research can add samples composed of different citizens, which will increase the authenticity of the research. In addition, the definition of social cohesion has six different dimensions, and this study only focuses on the degree of dependence, cooperation and solidarity between members of a society, which also has certain limitations.

## Data availability statement

The raw data supporting the conclusions of this article will be made available by the authors, without undue reservation.

## Author contributions

JF: conceptualization, methodology, and writing—original draft preparation. WL: investigation and writing—original draft preparation. HZ: editing. All authors contributed to the article and approved the submitted version.
